# Lack of replication of interactions between polymorphisms in rheumatoid arthritis susceptibility: case–control study

**DOI:** 10.1186/s13075-014-0436-x

**Published:** 2014-09-27

**Authors:** Aida Ferreiro-Iglesias, Manuel Calaza, Eva Perez-Pampin, Francisco J Lopez Longo, Jose L Marenco, Francisco J Blanco, Javier Narvaez, Federico Navarro, Juan D Cañete, Arturo R de la Serna, Isidoro Gonzalez-Alvaro, Gabriel Herrero-Beaumont, Jose L Pablos, Alejandro Balsa, Benjamin Fernandez-Gutierrez, Rafael Caliz, Juan J Gomez-Reino, Antonio Gonzalez

**Affiliations:** Laboratorio Investigación 10 and Rheumatology Unit, Instituto de Investigacion Sanitaria, Hospital Clinico Universitario de Santiago, Travesia Choupana, s/n, Santiago de Compostela, 15706 Spain; Rheumatology Unit, Hospital Universitario Gregorio Marañon, Calle Doctor Esquerdo, 46, Madrid, 28009 Spain; Rheumatology Unit, Hospital Universitario de Valme, Av de Bellavista, s/n, Sevilla, 41014 Spain; Rheumatology Unit, INIBIC-CH Universitario A Coruña, As Xubias, 84, A Coruña, 15006 Spain; Rheumatology Department, Hospital Universitario de Bellvitge, Feixa Llarga, s/n, Barcelona, 08907 Spain; Rheumatology Unit, Hospital Universitario Virgen Macarena, Avd. Dr. Fedriani, 3, Sevilla, 41009 Spain; Rheumatology Department, Hospital Clinic and IDIBAPS, Villarroel, 170, Barcelona, 08036 Spain; Rheumatology Unit, Hospital Santa Creu e San Pau, Carrer de Sant Quintí, 89, Barcelona, 08025 Spain; Rheumatology Service, Instituto de Investigación Sanitaria Hospital La Princesa, Hospital Universitario de La Princesa, Calle de Diego Leon, 62, Madrid, 28006 Spain; Rheumatology Unit. Fundación Jiménez Díaz, Avda. Reyes Católicos, 2, Madrid, 28040 Spain; Servicio de Reumatologia, Instituto de Investigación Hospital 12 de Octubre, Avda de Córdoba, s/n, Madrid, 28041 Spain; Rheumatology Unit, Hospital La Paz, Paseo de la Castellana, 261, Madrid, 28046 Spain; Rheumatology Unit, Hospital Clínico San Carlos, Calle Profesor Martín Lagos, s/n, Madrid, 28040 Spain; Hospital Vírgen de las Nieves, Av de las Fuerzas Armadas, 2, Granada, 18014 Spain; Department of Medicine, Universidad de Santiago de Compostela, Travesia Choupana, s/n, Santiago de Compostela, 15706 Spain

## Abstract

**Introduction:**

Approximately 100 loci have been definitively associated with rheumatoid arthritis (RA) susceptibility. However, they explain only a fraction of RA heritability. Interactions between polymorphisms could explain part of the remaining heritability. Multiple interactions have been reported, but only the shared epitope (SE) × protein tyrosine phosphatase nonreceptor type 22 (*PTPN22*) interaction has been replicated convincingly. Two recent studies deserve attention because of their quality, including their replication in a second sample collection. In one of them, researchers identified interactions between *PTPN22* and seven single-nucleotide polymorphisms (SNPs). The other showed interactions between the SE and the null genotype of glutathione *S*-transferase Mu 1 (*GSTM1*) in the anti–cyclic citrullinated peptide–positive (anti-CCP^+^) patients. In the present study, we aimed to replicate association with RA susceptibility of interactions described in these two high-quality studies.

**Methods:**

A total of 1,744 patients with RA and 1,650 healthy controls of Spanish ancestry were studied. Polymorphisms were genotyped by single-base extension. SE genotypes of 736 patients were available from previous studies. Interaction analysis was done using multiple methods, including those originally reported and the most powerful methods described.

**Results:**

Genotypes of one of the SNPs (rs4695888) failed quality control tests. The call rate for the other eight polymorphisms was 99.9%. The frequencies of the polymorphisms were similar in RA patients and controls, except for *PTPN22* SNP. None of the interactions between *PTPN22* SNPs and the six SNPs that met quality control tests was replicated as a significant interaction term—the originally reported finding—or with any of the other methods. Nor was the interaction between *GSTM1* and the SE replicated as a departure from additivity in anti-CCP^+^ patients or with any of the other methods.

**Conclusions:**

None of the interactions tested were replicated in spite of sufficient power and assessment with different assays. These negative results indicate that whether interactions are significant contributors to RA susceptibility remains unknown and that strict standards need to be applied to claim that an interaction exists.

## Introduction

Progress in the genetics of complex diseases such as rheumatoid arthritis (RA) has been very rapid in the last decade [1]. Many loci have been discovered that are convincingly and reproducibly associated with susceptibility to these diseases. About 100 loci are associated with RA susceptibility at the genome-wide association study (GWAS) level [2]. However, the advance in genetics has also shown that there are still many remaining challenges. One that has been the focus of much discussion is the large fraction of the genetic component that is still unknown. This fraction has been dubbed as missing heritability that for most complex diseases is larger than 50% [3]. The exact magnitude of missing heritability is disputable because of ambiguity in the estimation of the total heritability that needs to be explained [4]. In a study estimating heritability from GWAS data, a method that is not affected by genetic interactions, but that is insensitive to low frequency causal loci, the 100 known non–human leukocyte antigen (non-HLA) loci account for 4.7% to 5.5% of RA heritability [2]. This fraction has been reported to be smaller (about 2.5%) in twin studies [5], which are susceptible to confounding by genetic interactions but sensitive to low-frequency loci [4]. In addition, the large heterogeneity of RA adds to the ambiguity of the magnitude of missing heritability, as shown by the different genetic contributions of HLA shared epitope (SE) alleles in anti–cyclic citrullinated peptide–positive (anti-CCP^+^) patients (18%) and in anti-CCP^−^ patients (2.4%) [6]. Therefore, the missing heritability is very considerable in RA independently of the method used to estimate total heritability and the subgroup of patients being considered.

A variety of hypotheses have been proposed to explain the missing heritability [3], and there are already some studies in which researchers are exploring them in RA and other complex diseases [5,7-9]. One of these hypotheses is very compelling from a biological point of view. In that hypothesis, molecules, cells or tissues interact, and the outcome is often not fully predictable on the basis of consideration of the isolated factors. These ideas apply also to genes, whose products interact with the products of other genes and with the environment in gene × gene and gene × environment interactions, respectively. These interactions have been studied for a long time in genetics, but research in complex diseases has introduced in this field a strong incentive for more study together with the many difficulties typical of these diseases [10-13].

Interactions are invoked as a possible explanation for missing heritability because the combined effect of two genes can be larger than the sum of their individual contributions. In addition, genes that are not associated with disease susceptibility when considered in isolation can be associated in the presence of a modifier allele of other gene. Therefore, part of the missing heritability can be hidden in the form of interactions of known loci, because their contribution is considered one by one, as well as in loci not yet identified, because researchers in most GWASs have searched for associations single-nucleotide polymorphism (SNP) by SNP. It is also likely that the discovery of interactions will be of great utility for understanding the mechanisms of disease and to find very sensitive steps for therapeutic intervention. This hypothesis has driven multiple efforts to define types of interaction and their meaning and interpretation, as well as to develop tools to study them. Thanks to these efforts, there has been notable progress with tools that can be used to assess the association of all pairs of SNPs in a GWAS [14], as well as with statistical tests for interaction with increased sensitivity [10,15,16]. There has been also considerable clarification of the definitions and interpretation of the different types of interaction [10,13].

Research in RA has had an important role in the recent progress in the study of interactions in complex diseases. It has led to the discovery of one of the most reproducible examples: interaction between HLA-DRB1 SE and protein tyrosine phosphatase nonreceptor type *22* (*PTPN22*) risk genotypes and with smoking in anti-CCP^+^ patients [17]. These interactions have been widely replicated [18-20] and also have introduced interaction as a departure from additivity, the less well-known type of interaction [21], to the complex disease genetics field. However, the progress in interaction analysis has not yet translated to frequent attempts to replicate previous findings; therefore, the multiple interactions in RA that have been reported in the main genetics and rheumatology journals [22-31] remain without confirmation.

We aimed to replicate sound studies of gene × gene interactions in the genetics of RA. We selected two studies that included both discovery of interaction and its replication in additional samples [22,23]. One of them included testing of many pairwise interactions, and therefore multiple tests, but the authors corrected for them by proceeding in four selection steps in three different sample collections [22]. This first study led to the identification of seven SNPs showing multiplicative interactions with *PTPN22* that passed the four filters. In the second study, the authors analyzed only a gene × gene interaction and included replication in a second collection of samples [23]. The null genotype of glutathione *S*-transferase Mu 1 (*GSTM1*) showed additive interaction with the SE carrier genotype in the comparison of anti-CCP^+^ and anti-CCP^−^ patients. Our study did not replicate any of these interactions in spite of its sufficient power and the use of additional powerful tests. Therefore, we still need to find out how to improve reproducibility of interaction studies before knowing whether they are a significant contribution to RA susceptibility.

## Methods

### Participants

DNA samples from patients with RA and healthy controls were obtained as described previously [32]. All participants were of European Spanish ancestry. Briefly, the study included a total of 1,744 patients classed with RA according to the 1987 American College of Rheumatology criteria [33] and 1,650 healthy controls. All recruiting centers applied a questionnaire that asked all participants about demographic data, including the origin of their progenitors. Those reporting a non-Spanish progenitor at any level were excluded. The Ethics Committee for Clinical Research of Galicia approved this study, and the ethics committees at the recruiting centers (listed in the Appendix) approved sample collection. All participants gave their written informed consent. Clinical data for the patients, including the anti-CCP and HLA-DRB1 genotypes of 736 patients, were extracted from their clinical records or from previous studies [20] (Table 1).

**Table 1:** **Clinical features of the patients with rheumatoid arthritis** ^**a**^

**Clinical characteristics**	**RA patients**
Women (%)	75.7
Median age at disease onset (IQR)^b^	47 (37 to 57)
Morning stiffness (%)	96.2
Arthritis in three or more joint areas (%)	99.7
Arthritis of hand joints (%)	99.3
Symmetric arthritis (%)	99.1
Rheumatoid nodules (%)^b^	20.3
Rheumatoid factor (%)	71.2
Erosions (%)	68.6
Sicca syndrome (%)	8.9
Interstitial pneumonitis (%)	2.7
Shared epitope (carrier %)^b^	55.0
Anti-CCP (%)^b^	67.3

^a^CCP, Cyclic citrullinated peptide; RA, Rheumatoid arthritis. ^b^Data were available for <85% of the patients: 1,349 for age of disease onset, 1,283 for rheumatoid nodules and 736 for shared epitope and anti-CCP antibodies.

### Genotyping assays

The RA-associated *PTPN22* SNP rs2476601 (R620W) and the seven SNPs that have epistasis with it according to Briggs *et al.* [22], and an insertion/deletion polymorphism of exons 4 and 5 of *GSTM1* determining the native and null alleles that interact with SE according to Mikuls *et al*. [23], were studied with single-base extension assays (SNaPshot Multiplex Kit; Life Technologies, Carlsbad, CA, USA) applied to the products of a multiplex PCR carried out with the KAPA2G Fast HotStart enzyme (Kapa Biosystems, Wilmington, MA, USA). Detailed protocols, as well as the sequences of primers and probes used for these assays, are available upon request. Only the *GSTM1* polymorphism was uncommon, because, as in previous studies [23], it did not allow detection of heterozygous participants; only null homozygotes could be distinguished from carriers of native alleles. Quality control procedures used included manual revision of results, call rate >90% for each polymorphism, fit of genotype frequencies with Hardy-Weinberg equilibrium (HWE) (*P* >0.05), reproducibility (>99%) tested by regenotyping 10% of the samples, and comparison of allele frequencies with those in HapMap and with those reported in other studies.

### Statistical analyses

Allelic association of each polymorphism with RA susceptibility was assessed with χ^2^ tests. Replication of previously reported interactions was attempted first with the same statistical analysis used in the original study and afterward with alternative approaches. In this way, the multiplicative interaction between *PTPN22* rs2476601 and each of the other seven SNPs described by Briggs *et al.* [22] was tested as the coefficient of the multiplicative interaction term of a logistic regression model that included the *PTPN22* SNP and the interacting SNP coded according to a dominant model of their minor alleles. This coefficient is equal to the ratio of odds ratios of the interaction (ROR_i_). This analysis was done by comparing RA patients with controls; also, anti-CCP^+^ patients were compared with healthy controls, because most patients in the Briggs *et al*. study were anti-CCP^+^. All the previous analyses were performed with *STATISTICA* software (StatSoft, Tulsa, OK, USA). In turn, interaction analysis between the *GSTM1* null genotype and the SE was performed as described by Mikuls *et al.* [23]. That is, we compared anti-CCP^+^ patients with anti-CCP^−^ patients following a dominant model for the *GSTM1* native and *HLA-DRB1* SE alleles. The parameter analyzed was the attributable proportion (AP) to the interaction [21]. When AP is different from zero, it indicates the proportion of the association of the two loci that is attributable to departure from additivity. This analysis was done in R [34] using the Hosmer and Lemeshow approach [35].

The alternative approaches for analysis of the interaction included interchanging the analyses and other complementary tests—that is, testing the interactions used by Briggs *et al.* for departure from additivity and testing the relationship between the *GSTM1* null genotype and SE as a multiplicative interaction. These tests were complemented with others. First, we tested multiplicative interactions with the same three-term logistic regression model, but with the polymorphisms coded according to an additive genetic model (codes 0, 1 and 2 for the common allele homozygote, the heterozygote and the minor allele homozygote, respectively). Second, we used LRASSOC software to cover a range of saturated genetic models with and without interactions [36]. These models are built as logistic regression models, and the best model is selected by using Akaike’s Information Criterion (AIC), which is more sensitive than a significant difference between models. Models having an AIC difference <2 are not meaningfully different. Third, we performed two tests that, according to recent comparative studies, are the most powerful for detecting multiplicative interaction between two loci in a series of scenarios, provided that the two loci are in linkage equilibrium [15,16]. The first was the chi-squared Pearson statistic (T_Pearson_) in cases for independence of two loci [15]. The second was the adjusted Wu statistic for gametic phase disequilibrium in case-only analysis (T_AWu-co_) [16]. Power analysis was done applying the formula for power of ROR_i_ [37].

## Results

Genotyping assays for one of the SNPs (rs4695888, selected for its reported interaction with *PTPN22*) failed. The remaining polymorphisms were successfully assayed in 99.9% of the Spanish samples. Genotypes of all them fitted HWE (*P* <0.05). Only *PTPN22* rs2476601 of the SNPs from Briggs *et al.* was significantly associated with RA (OR = 1.53, 95% CI = 1.29 to 1.80, *P* = 4.61 × 10^−7^) (Table 2). Lack of association of the other SNPs was also the result in a previous report [22]. The *GSTM1* null genotype, in turn, was not different between anti-CCP^+^ and anti-CCP^−^ RA patients (Table 2, lower rows), whereas the SE was clearly more common in the anti-CCP^+^ patients than in the anti-CCP^−^ patients. These results are similar to those previously reported by Mikuls *et al.* [23].

**Table 2:** **Association of polymorphisms with rheumatoid arthritis or anti–cyclic citrullinated peptide–positive rheumatoid arthritis** ^**a**^

	**RA patients**		**Controls**			
**SNP**	**Genotype counts**	**MAF**	**Genotype counts**	**MAF**	***P***	**OR (95% CI)**
*PTPN22* rs2476601	1,383/325/35	0.11	1,405/235/10	0.08	4.61 × 10^−7^	1.53 (1.29 to 1.8)
rs7726839	1,012/631/100	0.24	959/592/88	0.23	ns	1.02 (0.91 to 1.14)
rs12573019	1,302/405/37	0.14	1,264/359/27	0.13	ns	1.11 (0.97 to 1.28)
rs1168587	652/830/260	0.39	612/762/276	0.40	ns	0.97 (0.87 to 1.05)
rs1895535	1,602/138/4	0.04	1,503/137/7	0.05	ns	0.91 (0.72 to 1.15)
rs7200573	940/687/116	0.26	890/633/127	0.27	ns	0.97 (0.87 to 1.08)
rs11865624	1,532/201/10	0.06	1,424/221/3	0.07	ns	0.92 (0.76 to 1.11)
	**Anti-CCP** ^**+**^ **patients**		**Anti-CCP** ^**−**^ **patients**			
	**Positive/negative**		**Positive/negative**			
SE	279/174^b^		116/153^b^		1.69 × 10^−6^	2.11 (1.56 to 2.88)
*GSTM1*	402/376^c^	–	186/192^c^	–	ns	1.10 (0.86 to 1.41)

^a^Comparison of patients with rheumatoid arthritis (RA) and healthy controls for the single-nucleotide polymorphisms (SNPs) from Briggs *et al.* [22] in the upper rows and comparison between anti–cyclic citrullinated peptide–positive (anti-CCP^+^) and anti-CPP^−^ RA patients for shared epitope (SE) and glutathione *S*-transferase Mu 1 (*GSTM1*) in the lower rows. MAF, Minor allele frequency; ns, Not significant; *PTPN22*, Protein tyrosine phosphatase nonreceptor type 22. ^b^Positive, SE carrier; negative, SE non-carrier. ^c^Positive, native carrier; negative, null homozygote.

### Lack of replication of epistasis with *PTPN22 rs2476601*

We tested the reported epistasis between six SNPs and the *PTPN22* rs2476601 SNP according to the original model: multiplicative interaction (evaluated as the interaction term of a logistic regression or its equivalent ROR_i_ between the minor alleles following dominant inheritance [22]. These analyses did not replicate any of the six instances of epistasis (Table 3). Lack of replication was not attributable to lack of power, because the *post hoc* type II errors (β) of missing results of a magnitude as the reported by Briggs *et al*. [22] at the final stage of their analyses were <0.20, which is conventionally considered sufficient (corresponding to statistical power of 0.80). The SNP with less power at the ROR_i_ term was rs7726839 with *post hoc* β =0.136, whereas all others had *post hoc* β <0.071.

**Table 3:** **Lack of replication of epistasis** ^**a**^ as the described by Briggs *et al*. [22]

**SNP**	**ROR**_**i**_ **(95%** **CI)**	***P***	**SNP carriers**	**SNP noncarriers**	**Anti-CCP** ^**+**^ **patients**	
			**OR**_***PTPN22***_ **(95% CI)**	**OR**_***PTPN22***_ **(95%** **CI)**	**ROR**_**i**_ **(95% CI)**	***P***
rs7726839	1.08 (0.75 to 1.55)	0.70	1.56 (1.18 to 2.07)	1.45 (1.15 to 1.83)	1.23 (0.8 to 1.9)	0.34
rs12573019	0.74 (0.49 to 1.12)	0.15	1.19 (0.83 to 1.71)	1.61 (1.31 to 1.98)	0.63 (0.38 to 1.04)	0.07
rs1168587	0.84 (0.58 to 1.22)	0.35	1.40 (1.11 to 1.76)	1.68 (1.24 to 2.24)	0.92 (0.59 to 1.43)	0.71
rs1895535	0.66 (0.36 to 1.20)	0.17	1.02 (0.58 to 1.80)	1.56 (1.25 to 1.92)	0.84 (0.39 to 1.8)	0.66
rs7200573	1.14 (0.8 to 1.63)	0.47	1.60 (1.24 to 2.07)	1.40 (1.10 to 1.80)	1.26 (0.82 to 1.93)	0.28
rs11865624	1.15 (0.68 to 1.94)	0.59	1.69 (1.04 to 2.75)	1.47 (1.21 to 1.78)	1.35 (0.72 to 2.53)	0.36

^a^Data show lack of replication between the six single-nucleotide polymorphisms (SNPs) and the *PTPN22* rheumatoid arthritis (RA) locus in the whole set of RA patients and controls (left) and in the comparison of anti–cyclic citrullinated peptide–positive (anti-CCP^+^) patients with controls (last two columns on the right). *PTPN22*, Protein tyrosine phosphatase nonreceptor type 22; ROR_i_, Ratio of odds ratios of the interaction.

**Figure 1:**
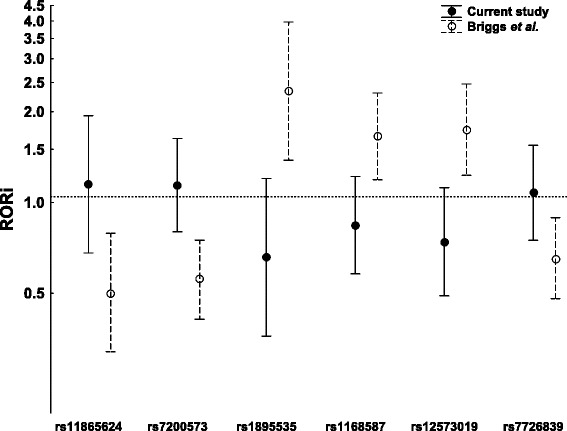
**Comparison of the multiplicative interaction terms (ratio of odds ratios of the interaction) from the present study and from Briggs** ***et al.*** **[** 22 **].** None of the 95% CIs from Briggs *et al*. cross the 1.0 line, showing that they are significant. All the CIs from the present study cross this line and show a nominal opposite direction. ROR_i_, Ratio of odds ratios of the interaction.

A lack of significant epistasis was observed, both with all patients and with anti-CCP^+^ patients. The lack of replication of epistasis was not dependent on the type of specific test or interaction model, because we tested the six SNPs for multiplicative interaction with the *PTPN22* rs2476601 SNP using several methods and none was positive (not shown). These tests included different genetic models in addition to the dominant one explored in the original report [22]. They also included the two tests reported as the most powerful in recent comparative studies [15,16]. In addition, we tested the six SNPs for interactions with *PTPN22* in the alternative framework for interaction, as a departure from additivity, and obtained similar results. We should note that, in addition to not showing significant association, the ROR_i_ of each of the six SNPs showed a nominal change in the opposite direction to the previously reported data (Figure 1).

### Lack of interaction between the null genotype of *GSTM1* and the shared epitope

**Figure 2:**
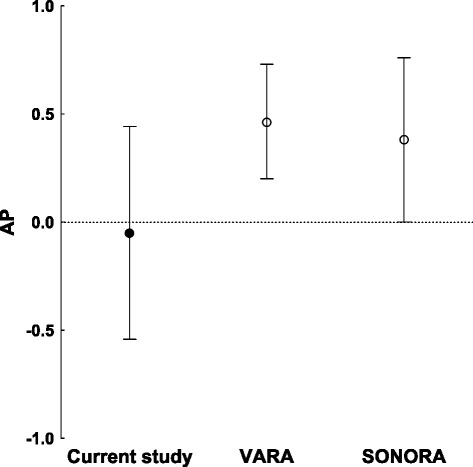
**Comparison of attributable proportions to interactions.** Graph depicts the attributable proportions to the interaction (APs) between the *GSTM1*-null genotype and shared epitope in anti–cyclic citrullinated peptide–positive rheumatoid arthritis patients in the present study and the two sample collections (Veterans Affairs Rheumatoid Arthritis (VARA) registry and Study of New-Onset Rheumatoid Arthritis (SONORA) sample set) included in the Mikuls *et al.* study [23].

Interaction between the null genotype of *GSTM1* and that carrying the SE has been described as a significant additive interaction in comparisons between anti-CCP^+^ and anti-CCP^−^ patients with RA [23]. In our samples, there was no evidence of this interaction, as the AP was not significantly different from zero (AP = −0.05, 95% CI = −0.54 to 0.44). None of the additional tests performed for deviations from additivity (relative excess risk due to interaction RERI and synergy [SI]) were significant, and neither were the tests used to assess multiplicative interactions (not shown), which were the same as those described in the previous subsection. In exploratory analysis, the OR for the patients with the *GSTM1* native genotype and SE was identical to the OR for the patients with the *GSTM1* null genotype and SE, reflecting the lack of increased association when the two risk genotypes were present (Table 4). Lack of sufficient power for replication was unlikely, because the sample sizes of the two collections of samples used by Mikuls *et al.* [23] were slightly smaller than those available to us (703 from the Veterans Affairs Rheumatoid Arthritis (VARA) registry and 610 from the Study of New-Onset Rheumatoid Arthritis (SONORA) sample set, compared to 721 in the present study). We applied analysis of confidence intervals as a way to explore power issues [38,39], owing to the lack of an analytical approach for estimating the power of AP. This analysis showed that our results were sufficient to exclude an interaction as the observed in the VARA sample, because the APs of the two studies fall outside the respective CIs (Figure 2). In contrast, our results were uncertain (neither excluding nor declaring them equivalent) in relation to the interaction observed in the SONORA sample, because the AP of the SONORA sample was within the CI of our study but the AP of our study is outside the CI of the SONORA sample (Figure 2).

**Table 4:** **Analysis of interaction between** ***GSTM1*** **genotype and** ***HLA-DRB1*** **shared epitope carrier status in comparison of anti-CCP** ^**+**^ **with anti-CCP** ^**−**^ **rheumatoid arthritis patients** ^**a**^

***GSTM1*** **/SE**	**Anti-CCP** ^**+**^ **/anti-CCP** ^**−**^ **(** ***n*** **)**	**OR (95% CI)**
Native/SE^−^	84/79	Reference
Null/SE^−^	90/74	1.14 (1.77 to 0.74)
Native/SE^+^	130/54	2.26 (1.46 to 3.52)
Null/SE^+^	148/62	2.25 (1.46 to 3.44)

^a^CCP, Cyclic citrullinated peptide; *GSTM1*, Glutathione *S*-transferase Mu 1; *GSTM1* native, Carrier native; *GSTM1* null, Homozygote null; SE, Shared epitope.

## Discussion

None of the seven interactions analyzed was replicated. This is very significant because (1) they have been reported in large studies that included replication in independent samples [22,23], and (2) our study is of sufficient power, we performed analyses for multiple inheritance and interaction models and we used the most powerful tests [15,16]. Therefore, we think our results raise questions about these interactions and, more generally, that they show how the level of reproducibility reached in RA association studies has yet to be achieved in interaction studies. As a consequence, only the previously known SE × *PTPN22* interaction pertaining to anti-CCP^+^ RA patients remains independently validated among the gene × gene interactions [17].

We attempted to replicate the Briggs *et al*. study where the authors have considered the whole genome for interactions with *PTPN22* [22]. As such, the number of tests could be very large and led to many false positives, but the Briggs *et al*. study included four steps of filtering. In the first step, 512 multiplex RA families were studied with microsatellites covering the genome. This analysis led to suggestive evidence in 5 loci containing 10,589 SNPs of the GWAS platform. These SNPs were tested in a case–control analysis (*n* =908 cases/1,260 controls from the North American Rheumatoid Arthritis Consortium I (NARAC-I)) for significant heterogeneity of *PTPN22* association in function of the SNP genotypes. The 665 SNPs showing significant heterogeneity were tested for multiplicative interaction in the subset of the NARAC-I sample with a more uniform Northern European ancestry. In this subset of 677 patients and 750 controls, the number of SNPs showing significant interaction was 449. These SNPs were tested again in an additional set of 947 patients and 1,756 controls (NARAC-II). Significant interactions were replicated for the seven SNPs we addressed in the present study. The least significant RORi showed a *P* =6.1 × 10^−3^ in the joint analysis of the two last case–control sets. Therefore, none of them was borderline significant; however, none was extremely significant, because the lower *P*-value was 1.5 × 10^−5^. In retrospect, it seems likely that the multiple filters were not sufficient to eliminate false-positive results although they reduced them. This makes it necessary to consider that joint analysis of all available samples with a higher threshold of significance could be more efficient for interaction analysis, as it is for association studies [40].

The Mikuls *et al*. study was hypothesis-driven, and the researchers examined a single gene × gene interaction [23]. Their aim was to explore the null genotype of *GSTM1* with regard to susceptibility to anti-CCP^+^ RA. The *GSTM1* null allele determines a deletion of the glutathione *S*-transferase gene, coding for a ubiquitous enzyme facilitating elimination of reactive oxygen species conjugated to glutathione. The null genotype has been associated with higher oxidative stress and increased risk for smoking-related inflammatory diseases, including RA [41-43]. Therefore, the hypothesis of Mikuls and colleagues was that the null genotype of *GSTM1* contributes to anti-CCP^+^ RA where smoking is known to increase risk. Analysis of the 703 patients of the VARA collection showed additive interaction between the *GSTM1* null genotype and SE when comparisons of the anti-CCP^+^ with the anti-CCP^−^ patients were made [23]. This result was replicated in the 610 patients of the SONORA study. Additive interactions are known between *PTPN22* and SE and between SE and smoking in the anti-CCP^+^ RA patients [17-20]. This type of interactions has been claimed to be more biologically relevant than multiplicative interactions [17,21], although there is a lot of discussion about the meaning of any of them [10,13]. Therefore, Mikuls *et al.* apparently found an additional piece of the network of interacting factors leading to anti-CCP^+^ RA [23]. However, it is noteworthy that they did not find interactions with smoking, which was another piece of the hypothesis and one that was relevant in other RA studies [41,43]. Our study did not replicate the interaction between *GSTM1* and SE in spite of the sufficient power and multiple tests for interaction.

It has been proposed that replicating gene × gene interactions is more difficult than replicating associations of individual SNPs. The additional difficulty is due in part to the study of polymorphisms that are not causal of the interaction, but only in linkage disequilibrium (LD) with the nongenotyped causal polymorphisms (markers or tags) [44]. The power of the replication is decreased by variation in the LD of the genotyped tag SNPs with the causal polymorphisms between the samples used for discovery and for replication. The reduction of power is proportional to the product of the decreasing LD between tag SNPs and the causal variants [44]. This factor is moderated in our study because *PTPN22* rs2476601, the SE alleles and the null genotype of *GSTM1* are causal polymorphisms, leaving no room for this effect in the interaction that was described by Mikuls *et al*. [23], and only for a linear decrease in power with the decrease in LD in the SNPs reported as interacting with *PTPN22* [22]. Another factor contributing to the lack of reproducibility is heterogeneity between studies. However, genetic heterogeneity does not have a large impact in RA, where most loci are shared between Europeans and Asians [2]. Therefore, it is uncertain if the lower frequencies of carriers of *PTPN22* rs2476601 and SE in our Spanish patients compared to patients in the previous studies conducted with participants of Northern European ancestry could have contributed to the discordant results (*PTPN22* risk allele carriers in our RA patients =20.7% vs. 27.8% and 27.3% in NARAC-I and NARAC-II, respectively, *P* <10^−4^ for both comparisons; and 54.6% of SE carriers in our RA patients vs. 75.5% and 71.1% in the VARA and SONORA samples, *P* <10^−15^ and *P* <10^−9^, respectively). Another type of heterogeneity that decreases reproducibility is clinical heterogeneity. In this regard, within the available information, we note only a much rarer smoking habit in our patients than in the patients in the Mikuls *et al.* study (20.6% of ever smokers among the RA patients we analyzed for this interaction vs. 79.8% and 65.1% in the VARA and SONORA cohorts, *P* <10^−93^ and *P* <10^−50^, respectively). Other differences, such as in the sex ratios, were observed only between our samples and one of the two collections used in the previous studies. Again, it is uncertain if the difference in smoking habit could impinge upon the results because no interaction between smoking and *GSTM1* null genotype was detected by Mikuls *et al.* [23]; however, such an interaction was reported in a different RA study [41].

The negative results we have obtained are discouraging because we selected for replication studies that were prominent in their methods and results, and because there is a conflict between biologic concepts that include interaction and the results of searching for its footprint in association studies. There are many other studies in which researchers have reported significant gene × gene interactions in RA [24-31], but only the *PTPN22* × SE interaction in anti-CCP^+^ patients has been independently replicated [17-20]. It has been a constant in the field to hope that better methods will allow discovery of prevalent interactions, but this hope has yet to be realized. Lack of reproducibility has also been observed between analyses of the same data with different approaches, as has happened with the Welcome Trust Case Control Consortium GWAS of seven complex diseases, including RA [14,45,46], and with the NARAC GWASs [47-50]. Therefore, it is not attributable only to insufficient power of the studies. Over time, it is becoming evident that discovery of interactions is a standing challenge for the genetics of complex diseases. Until a solution is found, researchers have no better choice than to increase demands on the threshold for significance and on independent replication for the assessment of interaction findings. It is to be expected that these measures will help to uncover the contribution that interactions have on the genetic components of RA and how these components work.

## Conclusion

No new gene × gene interactions in the susceptibility to RA have been independently replicated beyond the interaction between the SE and the risk allele of *PTPN22*. Our selection of two sound studies for replication led to negative results. The lack of interaction was found in spite of sufficient power and thorough analysis. This situation is unsatisfactory because interactions are widespread in biological systems, and they could help answer many questions, including the heritability not explained by known disease loci. Our negative results show that the contribution of genetic interactions to RA susceptibility still cannot be assessed and that strict standards for claiming interactions need to be applied.

## Appendix

The following ethics committees approved sample collection and this study: Comité Ético de Investigación Clínica de Galicia (currently, Comité Autonómico de Ética de la Investigación de Galicia), Comité Ético de Investigación Clínica del Instituto de Investigación Sanitaria Gregorio Marañón, Comité Ético de Investigación Clínica del Hospital Universitario Nuestra Señora de Valme, Comité Ético de Investigación Clínica de la Ciutat Sanitària i Universitària de Bellvitge, Comité Ético de Investigación Clínica del Hospital Universitario Virgen Macarena, Comité Ético de Investigación Clínica del Hospital Clínic i Provincial de Barcelona, Comité de Ética de la Investigación Clínica Fundación de Gestión Sanitaria del Hospital de la Santa Creu i Sant Pau, Comité Ético de Investigación Clínica del Hospital Universitario de La Princesa, Comité Ético de Investigación Clínica de la Fundación Jiménez Díaz, Comité Ético de Investigación Clínica del Hospital 12 de Octubre, Comité Ético de Investigación Clínica del Hospital Clínico San Carlos, and Comité Ético de Investigación Clínica del Hospital Universitario Virgen de las Nieves.

## Abbreviations

AIC: Akaike’s Information Criterion
Anti-CCP: Anti–cyclic citrullinated peptide
AP: Attributable proportion
CI: Confidence interval
GSTM1: Glutathione *S*-transferase Mu 1
GWAS: Genome-wide association study
HLA: Human leukocyte antigen
HWE: Hardy-Weinberg equilibrium
LD: Linkage disequilibrium
NARAC: North American Rheumatoid Arthritis Consortium
PTPN22: Protein tyrosine phosphatase nonreceptor type 22
RA: Rheumatoid arthritis
ROR_i_: Ratio of odds ratios of interaction
SE: Shared epitope
SNP: Single-nucleotide polymorphism
SONORA: Study of New-Onset Rheumatoid Arthritis
VARA: Veterans Affairs Rheumatoid Arthritis
